# Targeting Neuronal Alpha7 Nicotinic Acetylcholine Receptor Upregulation in Age-Related Neurological Disorders

**DOI:** 10.1007/s10571-025-01586-6

**Published:** 2025-07-16

**Authors:** Sharon Mariam Abraham, Sneha Suresh, Pragya Komal

**Affiliations:** 1 Department of Biological Sciences, Birla Institute of Technology and Science (BITS-Pilani), Hyderabad Campus, Jawahar Nagar, Kapra Mandal, Medchal District, Hyderabad, 500078 Telangana India; 2 Department of Biology, Indian Institute of Science Education and Research (IISER), Tirupati, 517507 Andhra Pradesh India

**Keywords:** Vitamin D3, Vitamin D receptor, Alpha7 nicotinic acetylcholine receptor

## Abstract

The multifunctional roles of alpha7 nicotinic acetylcholine receptors (α7nAChRs), ranging from cognitive enhancement, neuroprotection, and anti-inflammatory action, credit tagging this receptor as “unique” among the cholinergic receptor family. The uniqueness of α7nAChRs in neuronal function and communication lies in their high calcium permeability among the cholinergic receptor family. The ionotropic function of α7nAChRs is governed by protein kinases’ post-translational modification (PTMs), which alter their expression and function, affecting neuronal communication. A decrease in the ionotropic function of α7nAChRs and its downstream signaling pathways is observed across many neurological disorders. The loss of α7nAChRs, decreased cholinergic function, and increased acetylcholinesterase levels are commonly associated with neuronal degeneration, cognitive impairment, and decreased memory function. An extensive body of evidence suggests the cognitive benefits of simple nutraceutical supplementation, Vitamin D3 (VD), in many neurological disorders (Skv et al. in Mol Neurobiol 61:7211–7238, 2024). The present review will, however, focus on recent and past evidence deciphering the unique properties of α7nAChRs crucial for brain function. We have also emphasized on the therapeutic benefits of VD supplementation in restoring cholinergic neurotransmission and α7nAChRs expression in various neuropsychiatric and neurological disorders.

**Graphical Abstract:**

An overview of the importance and the therapeutic potential of α7nAChRs. α7nAChRs play a pivotal role in the maintenance of synaptic plasticity, cognitive enhancement, and neuroprotection. α7nAChR’s activation or restoration results in enhanced memory, cognitive restoration, anti-inflammatory effects, and neuroprotection in neuropathological states.

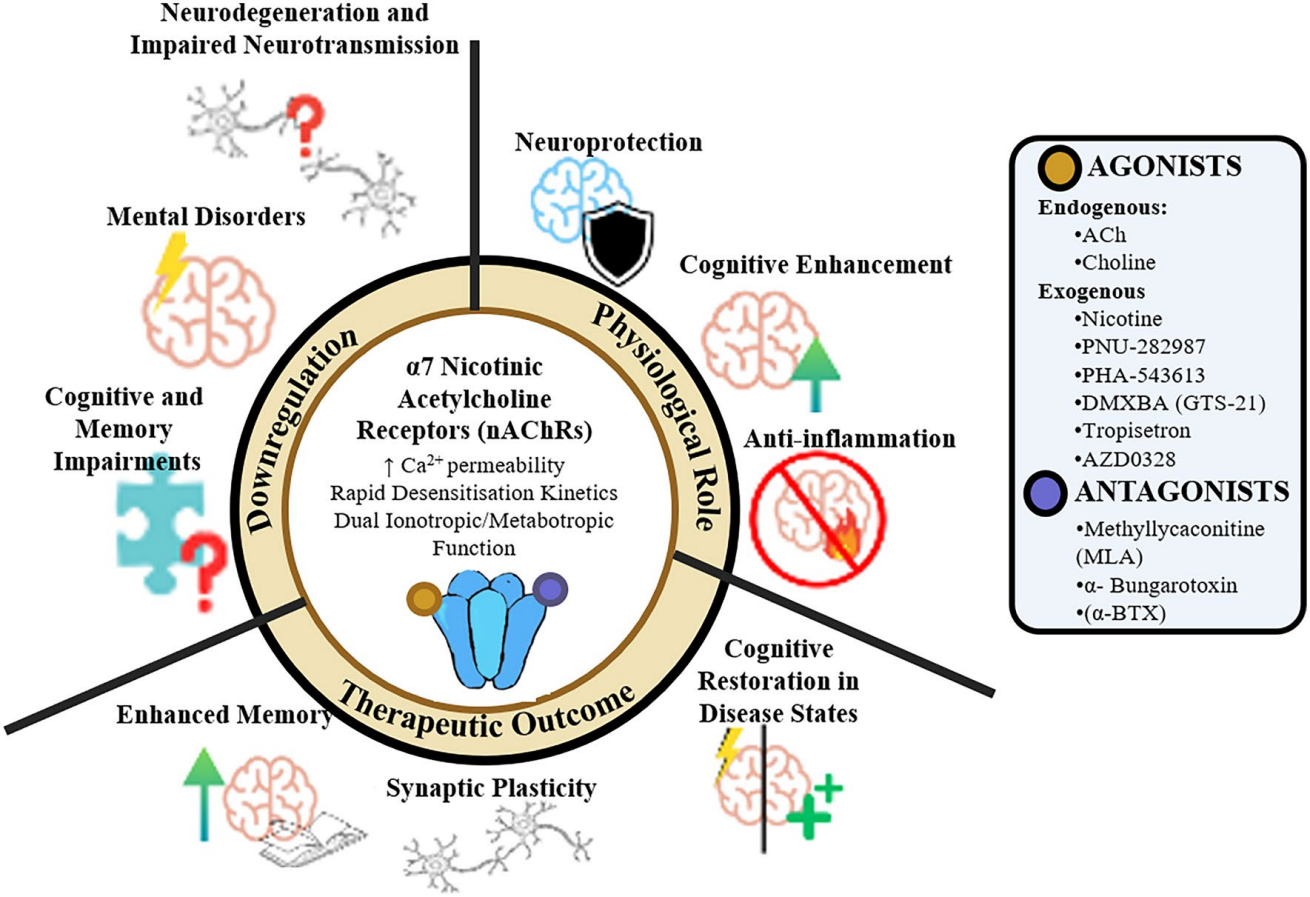

## Introduction

Mental illnesses experienced by patients suffering from depression, anxiety, and other neuropsychiatric disorders like schizophrenia (SCZ) affect cholinergic neurotransmission in regions of the brain vital for memory, cognition, attention, and perception (Raedler and Tandon 2006; Perez-Lloret and Barrantes 2016; Caton et al. 2020; Mahmoudi et al. 2023; Darrau et al. 2024; Yan et al. 2024). Evidence from neurobiological and genetic studies indicate that compounds targeting alpha7 nicotinic acetylcholine receptors (α7nAChRs) activation provide some benefits in various neuropsychiatric and neurodegenerative disorders (OLincy et al. 2006; OLincy and Freedman 2012; Sinha et al. 2020; Manetti et al. 2023; Abdel-Magid 2024) (Table 1). Cholinergic neurotransmission operates through nAChRs, and its impairment is reported in Parkinson’s disease (PD), Alzheimer’s disease (AD), SCZ, Huntington’s disease (HD), anxiety disorders, bipolar disorder, dementia, and depression (Raedler and Tandon 2006; Scarr et al. 2013; Perez-Lloret and Barrantes 2016; Papke and Horenstein 2021; Lee and Hung 2023; Mahmoudi et al. 2023; Ranglani et al. 2024). α7nAChRs appear as a key target for drug development aimed at improving treatments for these disorders, primarily due to their relatively high calcium permeability (Papke and Horenstein 2021).

**Table 1:** Summary of α7 nAChR-targeting compounds evaluated in different models of neurodegenerative, psychiatric, and cognitive disorders.

Sl. No	Disease	Compound	Type of compound	Model (in vivo*/ *in vitro)	Mode of action	Neuroprotective effect	References
1	Alzheimer’s disease	3-[(2,4-dimethoxy)ben zylidene]-anabaseine dihydrochloride (DMXBA/ GTS-21)	Selective partial α7 nAChR agonist	In-vitro: Primary culture of rat microglia treated with synthetic human Aβ42 hydrochloride In-vivo: Hemizygous APdE9 mice expressing chimeric mouse/human APPswe, DMXBA (1 and 5 mg/kg/day for 25 days)	Long- term administration: α7 nAChRs stimulation ↑↑ Aβ phagocytosis via calcium-regulated CaM-CaMKII and Rac1 signaling pathways which led to cognitive improvement	↑↑ acquisition, spatial cognition and memory retention in MWM task DMXBA (5 mg/kg) ↑↑ microglial phagocytosis of fibrillar Aβ, particularly Aβ42, (more prevalent in plaques and the FA-extracted fraction) DMXBA (≥ 1 mM in SH-SY5Y cells and ≥ 1 mg/kg in solubilized brain fraction)-neuronal ↓ γ-secretase activity which ↓ Aβ generation	( Takata et al. 2018 )
2	Alzheimer’s disease	PHA-543613	Selective α7 nAChR agonist	Presenilin 1 (PS1) and presenilin 2 (PS2) cDKO mice: AD model (impaired γ-secretase activity, resulting in alterations in APP processing and subsequent neurodegenerative changes characteristic of Alzheimer’s disease.)	↑ expression in hippocampal α7 nAChR protein levels reduced in cDKO mice Reverse the decreased synaptic protein level of NMDAR GluN2A and GluN2B subunits, and that of AMPAR GluA1 and GluA2 subunits Rescued impaired hippocampus-related spatial and working memory Recovered reduced LTP and PTP	Restored reduced α7 nAChR protein levels in the hippocampus seen in cDKO mice Significant improvement in spatial working memory and spatial reference memory as shown in Y-maze spontaneous alternation test and the working memory version of the MWM Restored LTP and PTP at the hippocampal CA3-CA1 pathway, which are crucial mechanisms underlying memory ↑ synaptic protein levels of NMDAR (GluN2A and GluN2B) and AMPAR (GluA1 and GluA2) subunits in cDKO mice ↑ hippocampal neural activity, as indicated by restored hippocampal theta oscillations and theta-gamma PAC ↑ activation of the AKT/GSK-3β signaling pathway, important for neuronal survival was seen	( Lv et al. 2023 )
3	Alzheimer’s disease	PNU-282,987	Selective α7 nAChR agonist	APPswe/PS1G384A mice (AD) mice of either sex, 1.5 and 6 months of age): Alzheimer’s disease mouse model (APPswe/PS1G384A mice, overexpressing human amyloid precursor protein (APP) with the Swedish double mutation (K670N, M671L) and a mutant presenilin 1 (PS 1, G384A mutation) under the control of Thy-1 promoter)	↓ levels of nAChRs and ↑ levels of α7 nAChR-bound Aβ1–42 are early biomarkers for AD α7 or α7β2 is involved in APP processing, learning, memory, and inflammation	↑ episodic memory and cognitive function in NORT ↑ mitochondrial stability ↓ neuroinflammation by ↓ pro-inflammatory cytokines (IL-1β, IL-6, TNF- α) and ↑ anti- inflammatory cytokines like IL-10 ↓ Aβ accumulation and its binding to α7 nAChRs	( Lykhmus et al. 2024 )
4	Alzheimer’s disease	PNU-282,987 (3 mg/kg/day) and in comb. with Bethanechol (mAChR agonist) (30 days treatment)	Selective α7 nAChR agonist	Doxorubicin (3 mg/kg, i.p., 6 doses)-induced chemobrain rat model: mimicking neuroinflammatory PCD and GSK-3β- induced Tau hyperphosphorylation (observed in AD)	α7 nAChR activation reduced cognitive dysfunction, mitigated neuroinflammation, restored mitochondrial homeostasis and rescued Tau phosphorylation thereby suppressing different forms of PCD	Separate and concomitant action of the agonists led to many effects, including: 1. ↑ PSD-95 expression, dendritic spine density and volume: Rescued synaptic plasticity 2. ↑ BBB integrity and hippocampal tight junction protein expression: ↑ claudin-5 & occludin expression 3. ↑ p-GSK-3β Ser9: Further ameliorated Tau hyperphosphorylation @ Thr181 4. Rescued hippocampal neuroinflammation: ↓ p-NF-κB Ser536, ↓ TNF-α expression, and ↑ IL-10 expression. PNU- 282,987 alone was able to increase STAT3 Tyr705 phosphorylation 5. ↓ Hippocampal CA1 region microglial and astrocytic activation: PNU-282,987 or Bethanechol separately increased astrocytic process length and branching 6. Preserved mitochondrial ROS-neutralizing capacity: ↓ Membrane depolarization, preserved mitochondrial membrane potential, and ↓ mitochondrial swelling 7. ↓ Excessive mitochondrial fission (↓ p-Drp-1 Ser616): Maintained mitochondrial fusion factor (MFN1, MFN2, OPA-1) expression 8. Attenuated apoptosis **{**↑ PI3K, ↑ p-AKT, ↑ p-ERK, ↑ Bcl-2, ↓ Cleaved Caspase-3**}**, pyroptosis **{**↓ Cleaved GSDMD, ↓ IL-1β (NLRP3 unchanged)**}** and necroptosis **{**↓ p-RIPK1, **↓** p-RIPK3, ↓ p-MLKL (Bethanechol did not reduce p-MLKL)**}**	( Ongnok et al. 2024 )
5	Huntington’s disease	Vitamin D3 (VD, 500 IU/kg for 15 days)	Modulator	C57BL/6 mice were administered with 3-nitropropionic acid (3-NP), 25 mg/kg for 3 days to model HD symptoms	↓acetylcholinesterase (AChE) activity, ↑↑ α7 nAChR mRNA and protein expression and ↓↓ TCR-β subunit gene expression in cortex and striatum	VD administration restored cholinergic signaling by reducing AChE activity. ↓ pro- inflammatory cytokine levels- TNF-α and IL-6, ↓ NF-κB gene expression and ↓ oxidative stress was also seen	( Manjari et al. 2023 )
6	Huntington’s disease	Tropisetron (3 mg/kg/day,i.p) for 14 days)	High-affinity partial agonist	Rat model of 3-nitropropionic acid-induced Huntington’s disease [3-NP (10 mg/kg/day, i.p.) for 14 days]	Partially activates α7-nAChR, leading to a controlled receptor stimulation level. Triggers downstream pathways without overstimulation, ↓ risk of desensitization and other adverse effects	↑ mobility, mean speed, rearing frequency by 1-, 1.1- and 2.1-fold respectively in OFT ↑ grip strength and fall of latency by 1- and 3.6-fold in grip strength test ↓ blood vessel wall thickening, astrocytic infiltration and lymphocytic small focal aggregation ↓ striatal GFAP expression and immunoreactivity ↑ SDH, Ho-1 activities, ↑ Nrf2 expression and ↓ MDA content showing anti-oxidative role Hampered JAK2/NF-κB inflammatory axis (↓ p-JAK2 and p–NF–κB p65 expression), ↑↑ PI3-K/Akt signaling (↑ p-PI3K and p-Akt expression) and, ↓ expression of IL1β, and TNF-α contents displaying an overall anti- neuroinflammatory role ↑↑Bcl-2 expression, ↓ Bax (↑Bcl-2/Bax ratio) and proapoptotic caspase-3 levels indicating anti- apoptotic activity	( Rabie et al. 2024 )
7	Parkinson’s disease	ABT-107 (0.25 mg/kg/day) with osmotic minipump administration	High affinity α7 selective nAChR agonist	In-vivo*:* 6-OHDA induced unilateral lesions into the medial forebrain bundle of male Sprague–Dawley rats In vitro*:* Striatal synaptosomes from lesioned and vehicle-treated rats	ABT-107 improved motor deficits associated with nigrostriatal damage, likely through enhanced striatal dopaminergic function, including increased dopamine release and elevated dopamine transporter (DAT) levels in the lesioned striatum	Improved motor deficits in forepaw placement and adjusted stepping tests ↑↑ striatal dopamine transporter (DAT) levels in lesioned striatum, ↑↑ basal and nicotine-stimulated dopamine release from lesioned striatum Enhanced α4β2* and α6β2* nAChR-mediated dopamine release	( Bordia et al. 2015 )
8	Parkinson’s disease	Nicotine (0.5 or 1 mg/kg)/ day for 7 weeks	α7 nAChR agonist	In-vitro*:* Primary mid brain astrocyte cultures of C57BL/6 newborn mice (1–2 days old) In vivo*:* Chronic MPTP (1-methyl-4-phenyl-1,2,3,6-tetrahydropyridine) intoxication to model progressive loss of dopaminergic neurons as seen in PD	α7nAChR activation by nicotine- Invitro- Inhibited astrocyte apoptosis induced by oxidative stress (H_2_O_2_), prevented the loss of mitochondrial membrane potential (*Ψm*), inhibited the cleavage of caspase-9, inhibited H₂O₂-induced GDNF downregulation In-vivo:- ↓ MPTP induced behavioral impairment and improved motor coordination, protected dopaminergic neurons against degeneration, inhibited astrocytes and microglia activation in SNpc, blocked MPTP-induced GDNF downregulation in the striatum and reversed the loss of TH-positive dopaminergic neurons	Nicotine administration protects astrocytes from H_2_O_2_-induced apoptosis by stabilizing mitochondrial function and inhibiting mitochondrial apoptotic pathway. It also plays a role in dopaminergic neuron degeneration in SNpc, inhibits dysfunctional astrocytic and microglial activation, reduces neuroinflammation, mitigates oxidative stress and rescues GDNF downregulation	( Liu et al. 2015 )
9	Hemiparkinsonism	3-[(2,4-dimethoxy)benzylidene]-anabaseine dihydrochloride (DMXBA/ GTS-21)	Functionally selective α7 nAChR agonist	Rat 6-OHDA-induced hemi parkinsonian model	Enhances α7 nAChR expression in dopaminergic neurons and microglia, reducing glial activation (including microglial neuroinflammation) and promoting dopaminergic neuroprotection in the substantia nigra, which is key in mitigating Parkinsonism	DMXBA reduced glial activation and rescued dopaminergic neurons by increasing α7nAChR expression. It also inhibited immunoreactivities to glial markers such as Iba1, CD68, and GFAP in the substantia nigra pars compacta of rats, suggesting a reduction in neuroinflammation	( Suzuki et al. 2013 )
10	Parkinsonism	PNU-282,987	Selective α7 nAChR agonist	α-SynWT-, α-SynA30P-, and α-SynE46K-N2a (Neuro- 2a) cells: in-vitro PD model	↑ α7nAChR expression and downstream pathways and ↑ autophagy of α-syn protein aggregates through TFEB- autophagy mechanism	↓ α-syn protein levels in all N2a cell lines after 48 h exposure ↑ TFEB promoter- luciferase activity, mRNA levels, and nuclear levels, ↑autophagy of α-syn aggregates& lysosomal biogenesis ↑LC3-II (autophagy activation marker) production and ↓ p62 expression (autophagy marker) levels	( Takizawa et al. 2024 )
11	Schizophrenia	Lu AF58801 (30 mg/kg; p.o.)	Selective and brain penetrant α7 nAChR PAM	Subchronic phencyclidine PCP- induced cognitive deficit model in Lister Hooded rats	Lu AF58801 potentiates the response of α7 receptors to ACh	Reversed PCP-induced cognitive deficit and improved cognitive performance in the NOR task	( Eskildsen et al. 2014 )
12	Schizophrenia	PNU-282987 (various doses upto 10 mg/kg; s.c.), RJR-2403 (various doses starting from 0.1 mg/kg), Donepezil (1 mg/kg)	PNU-282987 (Selective α7 nAChR full agonist), RJR-2403 (α4β2 nAChR agonist) Donepezil (AChE inhibitor)	Adult female hooded Lister rats	α7 receptor activation reverses delay-induced cognitive deficits in object recognition memory without affecting locomotor activity or total object exploration. Suggests that nicotinic receptor subtypes play an important role in forming and retrieving recognition memory, particularly in hippocampal-mediated mechanisms	Object recognition memory deficit can be induced in normal rats following a 6 h ITI, which was reversed by PNU-282987 (10 mg/kg; s.c.), RJR-2403 (0.1 mg/kg) and donepezil; suggesting precognitive effects of the drugs	( McLean et al. 2016 )
13	Schizophrenia	PNU282987, NS1738 alone and in comb. with atypical antipsychotic drug risperidone	PNU282987 (selective α7 agonist), NS1738 (α7 PAM)	Wistar rats used in CAR, FST, and microdialysis experiments and Sprague–Dawley rats used for NOR and electrophysiology experiments	α7 modulators were able to synergistically enhance the dopaminergic and glutamatergic neurotransmission, which contributed to working memory and cognitive deficits in SCZ	Combined effect of PNU282987 or NS1738 with risperidone significantly facilitated NMDA-induced currents in layer V/VI pyramidal cells of the mPFC, which may underpin improvements in cognition and working memory. Any drug alone had no effect In CAR test, both PNU282987 and NS1738 enhanced the antipsychotic effects of risperidone, NS1738 was more potent In NOR test, both PNU282987 and NS1738 ↑↑ recognition memory when used alone Both the modulators in combination with risperidone were able to enhance the dopamine release in the NAc (but not in mPFC), which may improve negative symptoms like anhedonia PNU282987 exhibited antidepressant-like effects in FST	( Marcus et al. 2016 )
14	Schizophrenia	PNU282987, SSR180711, NS1738, PNU120596	PNU282987 (full agonist), SSR180711 (partial agonist) NS1738 (PAM type I) and PNU120596 (PAM type II)	MAM developmental disruption model	α7nAChR activity modulation impacts the dopaminergic system in a state- dependent manner. enhances VTA dopaminergic (DA) neuron activity (particularly through localized activation in BLA). This modulation has a more significant impact under normal conditions (control) than in hyperdopaminergic (MAM rats) state	In control rats with BLA infusion: PNU282987 and SSR180711 ↑↑ dopamine (DA) neuron activity in the central VTA In control rats with systemic infusion: PNU120596 ↑↑ DA neuron activity in the medial VTA In MAM rats with BLA fusion: No significant changes, but SSR180711 infusion ↑ burst firing in vehicle-treated MAM rats In MAM rats with systemic infusion /vHipp infusion: PNU282987 and SSR180711 ↓↓ DA neuron population activity in lateral VTA compared to vehicle- treated rats. No significant changes in the medial or central VTA	( Neves and Grace 2018 )
15	Schizophrenia	A-582941 (1 mg/kg for 10 days)	α7-nAChR agonist	MK-801 induced mouse model of schizophrenia (sub-chronic, 0.2 mg/kg, 2 doses/day for 7 days ip., last dose was s.c)	↑↑ discrimination index in NORT, swimming time in platform area in MWM, social following behavior ↓↓ social avoidance and platform- finding latencies Administration had no effect on PPI, no rescue in sensorimotor gating deficit Repeated administration of α7-nAChR agonist leads to receptor upregulation, showcasing these pro-cognitive and prosocial effects	A-582941 improved social deficits and cognitive dysfunctions on visual and spatial memory, thus, having a stronger effect on negative and cognitive dysfunctions compared to drug Clozapine. Clozapine only improved social following behavior but had no effect on avoidance	( Unal et al. 2021 )
16	Schizophrenia	2-arylamino-thiazole-5-carboxylic acidamide derivatives 6–9 (6p and 7b)	Atypical type I PAM	MK-801 (NMDA antagonist, 0.1 mg/kg, i.p.) induced mouse model of schizophrenia	Activate α7 nAChRs by enhancing receptor response and delaying gating kinetics, prolonging receptor function. By maintaining/controlling receptor desensitization, they effectively prevent Ca2+ overloading and reduce the risk of cytotoxicity	Both 6p and 7b interact with a novel allosteric site, distinct from the conventional PNU-120596 binding pocket. The compounds were able to attenuate PPI impairment and rescue the auditory gating deficit in mice in a dose-dependent manner, aiding cognition	( Yang et al. 2024 )
17	Major Depressive Disorder (MDD)	PNU120596 (1 or 4 mg/kg, α7-nAChR PAM)	PNU120596 (1 or 4 mg/kg, α7-nAChR PAM) ANA12 (0.25 or 0.5 mg/kg, TrkB receptor antagonist) In combination- PNU120596 (1 mg/kg) + ANA12 (0.25 mg/kg)	LPS (1 mg/kg)- induced neuroinflammation and cognitive deficit- Major Depressive Disorder (MDD) Male C57BL/6 J mouse model. LPS administration results in ↑ BDNF and the NKCC1/KCC2 ratio and ↓ KCC2 expression in the hippocampus and prefrontal cortex by activating TrkB signaling, resulting in GABAergic- neurotransmission dysregulation	PNU120596- ↓↓ LPS-induced BDNF expression increment and NKCC1/KCC2 ratio and ↑ KCC2 expression ANA12- ↓ LPS- induced cognitive deficit and depressive like behaviors- ↓↓ spontaneous alternation in the Y-maze and ↑↑ immobility duration in TST and FST Coadministration of subthreshold doses showed cognitive benefits	α7 nAChRs are involved in anti-inflammatory cholinergic pathways that modulate microglial activation, the primary source of neuroinflammation in the brain PNU120596 prevented the LPS-induced depressive-like behavior by likely ↓↓ neuronal excitability via targeting microglial α7 nAChR in the hippocampus and prefrontal cortex. ↓↓ neuroinflammation and restored chloride ion balance via regulation of NKCC1/KCC2 expression, potentially alleviating depressive-like behaviors	( Alzarea et al. 2024 )
18	Cognitive recognition deficit	CCMI and PNU-120596	CCMI (type I PAM) and PNU-120596 (type II PAM)	Scopolamine induced AD-like cognitive recognition deficit model in rats (Male Sprague–Dawley rats)	PAMs are usually able to enhance the activity of α7-nAChRs in the presence of the endogenous ligand, acetylcholine (ACh), by enhancing the receptor’s response to the ligand. In Alzheimer's disease (AD), there is progressive degeneration of cholinergic neurons, especially in the basal forebrain. Additionally, β-amyloid (Aβ) plaques bind to α7-nAChRs to form complexes, disrupting their function. This disruption can be prevented by α7-nAChR ligands like ACh. The use of AChEIs increases the amount of Ach in the synapse, allowing PAMs to enhance the activity of α7-nAChRs and restore cholinergic neurotransmission	On their own and in combination with standard AD drugs (acetylcholinesterase inhibitors (AChEIs- donepezil and galantamine, or the non-competitive NMDAR antagonist, memantine), these drugs rescued the cognitive deficit in NORT	( Potasiewicz et al. 2020 )
19	Cognitive deficit	BNC375	Type I α7 PAM	In-vivo models- 1. Scopolamine-induced cognitive deficit model of rat: NOR analysis Rat hippocampus: fEPSP recordings 2. Scopolamine-induced cognitive deficit model of rhesus monkey (*Macaca mulatta*): NOR analysis 3. Aged green monkey: Object retrieval detour task In vitro models- 1.Human embryonic kidney (HEK-293 T) cells and GH4C1 cells transfected with human α7nAChRs 2. Primary cultures of rat cortical neurons	↑ ACh-evoked currents through the α7 nACh receptor by enhancing receptor response to acetylcholine, without altering activation or desensitization kinetics	↑ cognitive function in scopolamine- induced deficits in rats and NHPs ↑ glutamatergic and GABAergic neurotransmitter cycling indicating increased energy metabolism in the brain ↑ LTP in hippocampal slices and anesthetized rats in dose- dependent manner, confirming effects on long- term synaptic plasticity	( Wang et al. 2020a, b )
20	Depression	NS-1738, PNU-120596 and PAM-2	NS-1738 (type I PAM), PNU-120596 and PAM-2 (type II PAM)	Naïve C57BL/6 J male mice were subjected to depressive tests like the FST and TST to induce depressive-like behavior	Both type I and type 2 PAMs, on chronic treatment, were able to induce anti-depressant-like activity mainly involving receptor potentiation but not by delaying/inducing desensitization or by neurotransmitter transporter blockade	NS-1738 (sub-chronic treatment, 7 days), PNU-120596 (sub-chronic treatment), and PAM-2 (chronic, 14 days) treatment showed antidepressant-like effects in FST and TST PAM-2 increased phosphorylation of mTOR, ERK1/2, GSK-3β, 3β and RSK-1 in the PFC and hippocampus, which may have aided the anti-depressant-like activity This activity was inhibited by MLA- indicating the anti-depressant effect of α7nAChRs	( Targowska-Duda et al. 2021 )

Compounds are organized by disease category and annotated with their pharmacologic type, experimental model, mechanism, and observed neuroprotective outcomes

nAChRs belong to the cys-loop family of ligand-gated ion channels, which include GABA_A_, 5-HT3, and glycine receptors, and contain a conserved pair of disulfide-bonded cysteines separated by 13 residues (Nashmi and Lester 2006; Yakel 2013; Komal et al. 2014, 2015b; Komal and Nashmi 2015a). Neuronal ionotropic receptors comprise eight different *α* subunits: α2–α7, α9–α10, and three other *β* subunits, namely β2–β4. A functional pentameric nAChR is formed by combining five divergent subunits, either only in α-subunits or in α- and β-subunits, with ligand-binding sites located between two α-subunits or between α- and β-subunits (Gotti and Clementi 2004; Gotti et al. 2009; Lindstrom 2010). Each subunit contains four transmembrane domains (M1–M4) with an extracellular amino (N-terminal) and carboxyl (C-terminal) termini (Nashmi et al. 2007; Shen and Yakel 2009; Komal et al. 2014, 2015; Noviello et al. 2021; ZHuang et al. 2022). The long cytoplasmic loop between M3-M4 contains putative phosphorylation sites for different protein kinases (Komal et al. 2014).

**Fig. 1:**
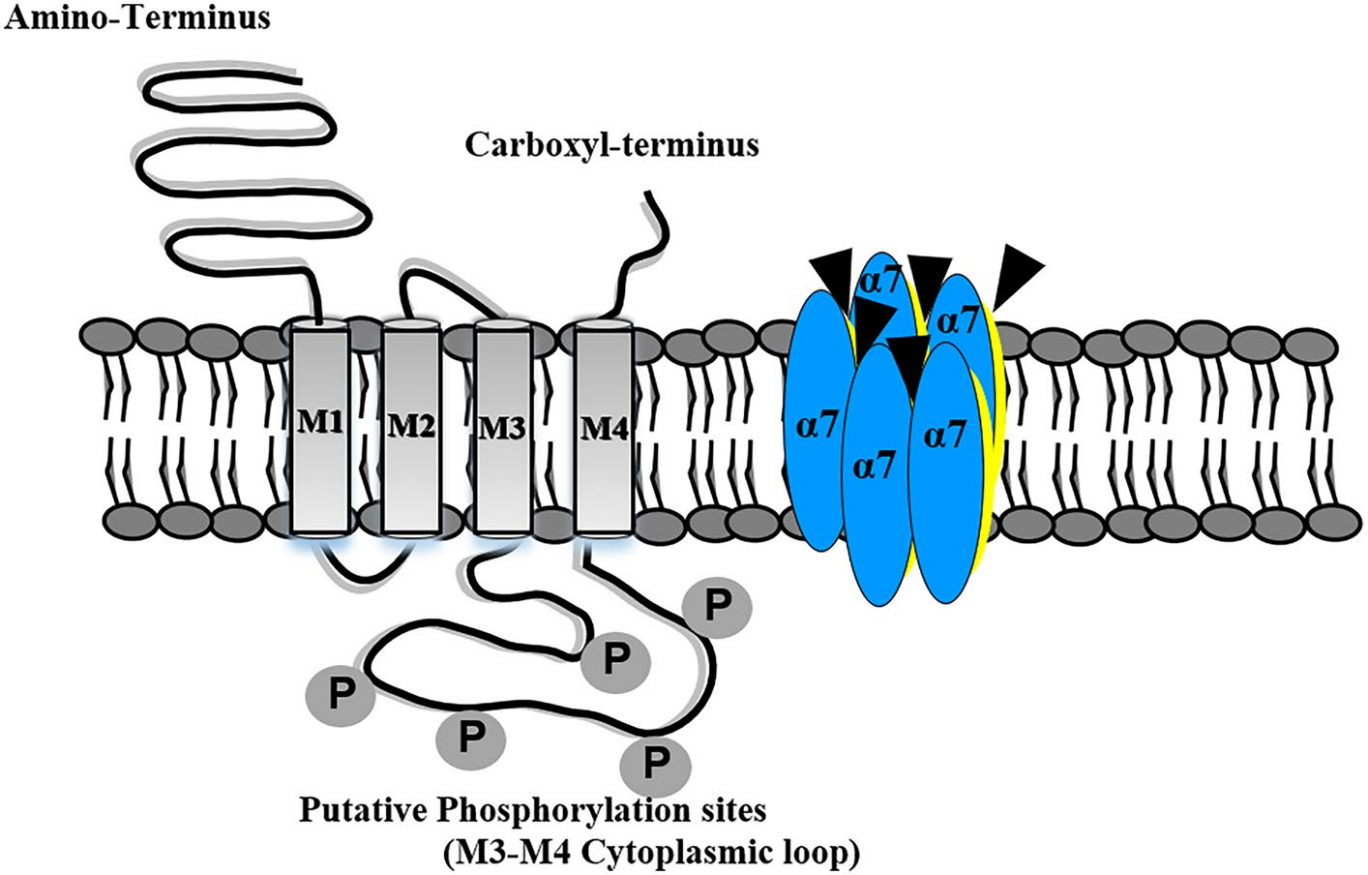
Diagrammatic representation of a single subunit of α7nAChR showing amino and carboxyl terminals. M3-M4 cytoplasmic loop of the channel contains putative phosphorylation sites for protein kinases like Src kinase and protein kinase A (PKA). A functional receptor is formed by combining five subunits (shown in blue) and contain five agonist binding sites (shown in black triangles) (Komal et al. 2014, 2015)

This large intracellular cytoplasmic loop is the region of greatest divergence within the nicotinic receptor family and provides a platform for macromolecular interaction with molecules like PSD-95 and scaffold protein (Leonard and Bertrand 2001; Valor et al. 2002; Neff et al. 2009; Sinclair and Kabbani 2023). It contains motifs and specific sites for receptor assembly, trafficking, and modification (Berg and Conroy 2002). The M4 helix is directly involved in receptor gating and function (da Costa Couto et al. 2020). M3 and M4 helices are separated by a large, variable intracellular loop that contains putative phosphorylation sites for serine/tyrosine kinases (Fig. 1) (Komal et al. 2014, 2015). On agonist binding, the receptor is known to undergo three allosteric conformations- closed, open, and desensitized (Gotti and Clementi 2004; Komal et al. 2011; Noviello et al. 2021). These transitions are reversible, and different ligands stabilize different receptor states: agonists initially stabilize the open state, whereas competitive antagonists preferentially stabilize the nAChR in a closed state, either the resting or desensitized configuration (Papke and Lindstrom 2020). The rate of desensitization and recovery also differs from one type of nAChR to another, with α7nAChRs exhibiting rapid desensitization kinetics (Komal et al. 2011; ZHuang et al. 2022). Thus, each nicotinic receptor subtype shows a wide array of distinct physiological and pharmacological properties (Nashmi and Lester 2006; Shen and Yakel 2009; Xiao et al. 2009; Komal et al. 2011; Picciotto et al. 2012; Renda et al. 2016).

α7nAChRs constitute the most abundant homopentameric cholinergic receptor, while α4β2* (*represents that other subunits may also be present) forms the most abundant high-affinity heteromeric nicotine binding receptor expressed in the mammalian central nervous system (CNS) (Nashmi and Lester 2006). α7nAChRs may not be purely homopentameric and are reported to exist as heteromeric channels in rodents and human brains (Wu et al. 2016; Letsinger et al. 2022). α7nAChRs opening occurs on the binding of the endogenous neurotransmitter acetylcholine (ACh), exogenous ligand nicotine, and by choline, the breakdown product of ACh (Alkondon et al. 1997; Komal et al. 2011; Hou et al. 2018). These receptors display a high affinity for α-bungarotoxin and low affinity to ACh and nicotine, unlike α4β2*nAChRs (Albuquerque et al. 2009; Komal et al. 2014, 2015). RIC-3 (Resistant to Inhibitors of Acetylcholinesterase), a transmembrane chaperone protein, promotes the maturation, assembly, and surface trafficking of α7nAChRs (Dau et al. 2013).

**Fig. 2:**
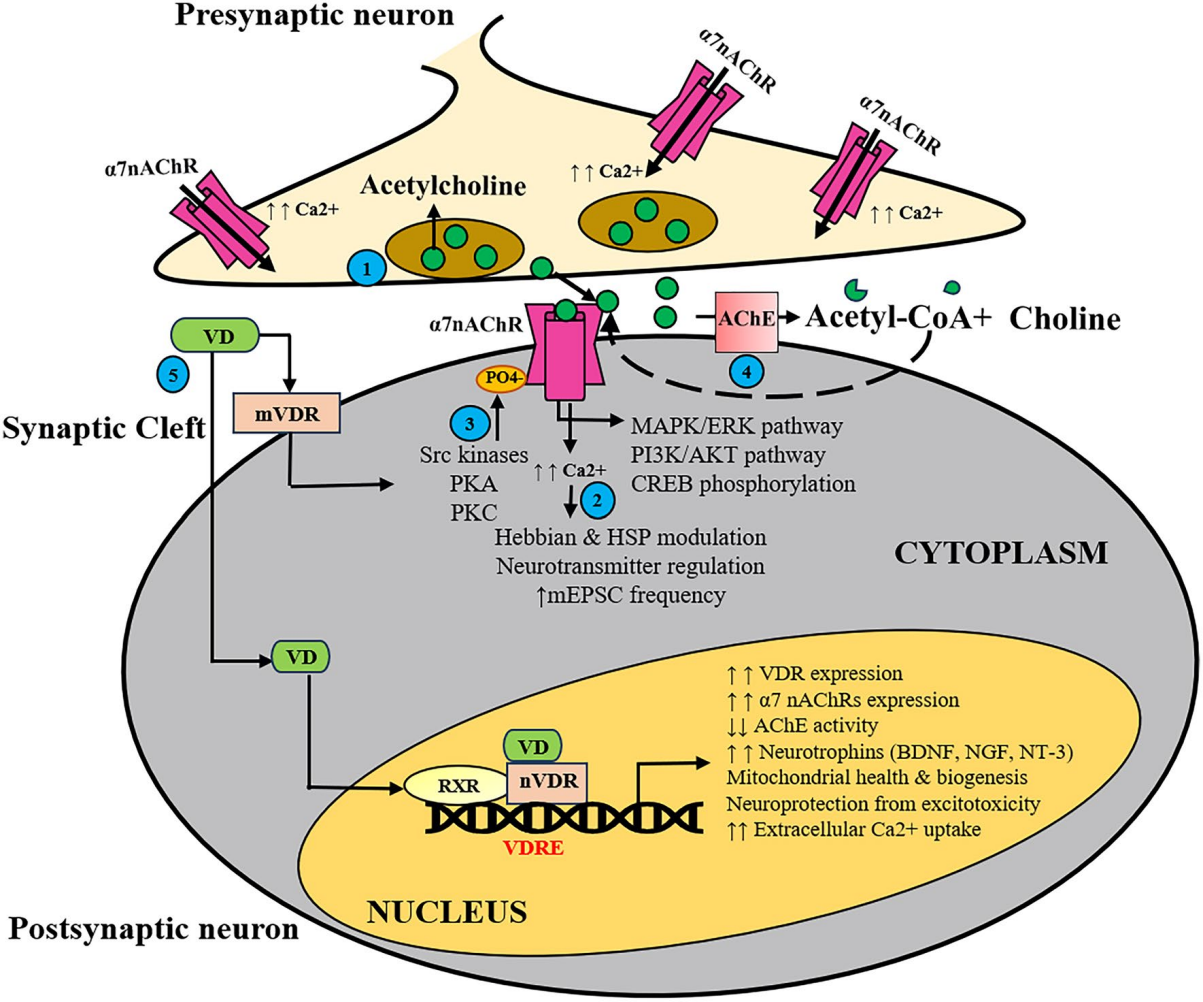
Schematic representation of α7nAChRs metabolism and neuroprotective effect of VD. Steps 1 to 4 illustrate the major steps of α7nAChRs activation from binding to the endogenous ligand, ACh, to its metabolism by AChE. (1) Acetylcholine released from presynaptic neurons activates α7nAChRs, acting as an agonist. (2) This activation increases Ca2+ permeability, leading to various neuromodulatory functions. (3) The receptor undergoes phosphorylation by various kinase enzymes, activating multiple signaling pathways. Additionally, various phosphatases (tyrosine phosphatase, serine–threonine phosphatase) boost α7nAChRs activity (not shown here). (4) Acetylcholine is metabolized by the acetylcholinesterase enzyme into acetyl-CoA and choline. (5) Vitamin D3 (cholecalciferol, VD), through VD receptors (VDRs), exerts various neuroprotective actions, including enhanced α7nAChR gene and protein expression and reduction in acetylcholine esterase (AChE) activity (Picciotto et al. 2012; Komal et al. 2022; Manjari et al. 2023; Skv et al. 2024). Presynaptic localization of α7nAChRs on different neurons and their high calcium permeability can directly cause neurotransmitter release and modulate neuronal excitability (Girod et al. 2000; Jones and Wonnacott 2004; SharMa et al. 2008). Abbreviations:*α7nAChR* alpha7 nicotinic acetylcholine receptor, *PO4-* phosphate ion, *Ca2+ * Calcium ion, *PKA* Protein kinase A, *PKC* Protein kinase C, *AChE* Acetylcholinesterase enzyme, *VD* Vitamin D3, *nVDR* Nuclear Vitamin D receptor, *mVDR* Membrane-bound Vitamin D receptor; RXR: Retinoid X receptor; VDRE: Vitamin D Response Element; BDNF: Brain-derived neurotrophic factor, *NGF* nerve growth factor, *NT-3* Neurotrophin-3, *HSP* Homeostatic synaptic plasticity, *mEPSC* Spontaneous miniature excitatory postsynaptic currents

The Ca2+ /Na+ permeability ratio of α7nAChRs is more than α4β2 nAChRs and almost equal to that of N-Methyl-D-Aspartate receptor type of glutamate (NMDA) receptors (Séguéla et al. 1993). This high calcium permeability of α7nAChRs is associated with metabotropic activity and regulates many calcium-mediated second-messenger signaling transduction pathways (Berg and Conroy 2002). An excellent review has recently elaborated on the dual ionotropic and metabotropic properties of α7nAChRs that affect calcium and cytoskeletal dynamics in a cell-specific manner (Sinclair and Kabbani 2023). The ligand-induced opening of α7nAChRs impacts several Ca2+ dependent signaling pathways, including kinase activation and regulation of gene transcription (Placzek et al. 2009; Komal et al. 2011, 2014, 2015; Noviello et al. 2021; Papke and Horenstein 2021; ZHuang et al. 2022; Sinclair and Kabbani 2023). Neuronal activity-dependent Ca2+ influx through nAChRs induces multiple intracellular signals that have essential roles in synaptic development, maintenance, and plasticity (Dani et al. 2001; Yakel 2013; Dani 2015; Letsinger et al. 2022; Nakamura et al. 2023). Another study by Koninck and Cooper demonstrated that α7nAChRs opening facilitated a direct activation of the calcium calmodulin kinase (CaM) pathway in cultured neonatal rat sympathetic neurons (De Koninck and Cooper 1995). An unconventional role of α7nAChRs was shown in microglial culture, where activation of this receptor by nicotine induced calcium (Ca2+) release from ryanodine receptors with stimulation of the inositol 1,4,5-trisphosphate (IP3) signal transduction pathway (Suzuki et al. 2006). The importance of the rise of intracellular calcium on α7nAChRs activation comes from another *in-vitro* study conducted in hippocampal cultured neurons (Dajas-Bailador et al. 2002). Nicotine-mediated stimulation of α7nAChRs led to another signal pathway activation that involved protein kinase A (PKA) and extracellular signal-regulated kinases (ERK1/2) (Fig. 2) (Dajas-Bailador et al. 2002). Calcium cations influx through direct opening of α7nAChRs are known modulators of cytosolic calcium homeostasis, neuronal function, and survival (Uteshev 2012). This cholinergic receptor has been shown to possess even greater relative Ca2+ permeability than the NMDA subtype of glutamate receptors (Séguéla et al. 1993). Thus, extensive evidence showed that the importance of calcium influx on opening of α7nAChRs which can mediate the initiation of a wide spectrum of intracellular signal cascades that, in turn, facilitate synaptic plasticity and memory formation (Berg and Conroy 2002; Shen and Yakel 2009; Phenis et al. 2020).

α7nAChRs are known to be operative throughout the brain, facilitating neurotransmitter release, cognition, attention, learning, and memory (Egea et al. 2015; Li et al. 2018; Caton et al. 2020; Piovesana et al. 2021; Nakamura et al. 2023; BaLi et al. 2024; Abbondanza et al. 2024). α7nAChRs have been a critical focus in current neurological and psychiatric research because dysregulated gene expression has been found in these conditions. Much evidence comes from preclinical studies conducted on rodent models (Lewis et al. 2017; Sinha et al. 2020; CoughLin et al. 2020; Wu et al. 2022; Abdel-Magid 2024). This review will discuss findings where restoration of α7nAChRs expression and function impacts cholinergic neurotransmitter dynamics in the CNS. We have tried to illustrate the pleiotropic functions contributed by α7nAChRs toward brain health.

## Importance of α7nAChRs in Synaptic Plasticity and Homeostatic Synaptic Plasticity

Earlier pioneers showed the vital role of the ACh and cholinergic neuronal network in cognition and cortical function (Krnjević et al. 1971; Aigner et al. 1987). Later, in 1994, Auerbach and Segal showed a direct link between ACh and cholinergic effects on synaptic transmission and synaptic plasticity in rat hippocampal slices (Auerbach and Segal 1994). In the CNS, cholinergic neurons release the ACh mainly from four regions of the mammalian CNS, namely, the brainstem, a group of thalamic nuclei, the striatum, and the nuclei present in the basal forebrain (Picciotto et al. 2012; Li et al. 2018; Ananth et al. 2023). The efficacy of cholinergic neurotransmission depends mainly on the activity and duration of the acetylcholine esterase (AChE) enzyme (Fig. 2). AChE cleaves ACh into acetyl-CoA and choline and terminates signaling in the cholinergic system (Picciotto et al. 2012).

The release of ACh shapes cortical function and cognition during wakefulness (Yang et al. 2013). The higher cognitive role of α7nAChRs mainly comes from a study performed at the glutamatergic synapses in the dorsolateral prefrontal cortex (dlPFC) that revealed a direct effect of α7nAChRs activation, leading to the excitation of NMDA receptors that facilitated working memory functions (Yang et al. 2013). The significance of α7nAChRs in cognition and memory function was confirmed by gene deletions that showed loss of this receptor caused a significant reduction in the synaptic expression of NMDA receptors and glutamatergic synaptic deficits in the mouse cortex (Lin et al. 2014). The activation of α7nAChRs in the PFC was reported to impact associative recognition memory (Sabec et al. 2018). Multiple studies show a neuromodulatory function of cholinergic transmission in the CNS, regulating the release of multiple neurotransmitters in neural circuit-specific manners that bring diverse behavioral effects through pre- and post-synaptically located nAChRs (Picciotto et al. 2012). Many studies have shown that ligand-induced opening of α7nAChRs affects glutamatergic and GABAergic neurotransmission in various regions of the CNS, like dlPFC, the cortex, the striatum, and the hippocampus (Girod et al. 2000; Buhler and Dunwiddie 2002; Berg and Conroy 2002; Matsubayashi et al. 2004; Pakkanen et al. 2005; Yang et al. 2013; Maex et al. 2014; Verhoog et al. 2016; Xu et al. 2021).

Parikh and colleagues showed the pivotal role of α7nAChRs in facilitating the cross-talk between glutamatergic-cholinergic signaling in the PFC. This function mediated by α7nAChRs was mandatory for cue detection and attentional performance (Parikh et al. 2010). Evidence highlighted the layer-specific expression of α7nAChRs in driving the activation of the excitatory and inhibitory neuronal networks in the mammalian PFC (Poorthuis et al. 2013). α7nAChRs were shown to be located in the glutamatergic inputs of layer V pyramidal neurons and layer II-III GABAergic interneurons of PFC, where they regulated respective neuronal activation (Poorthuis et al. 2013). Other studies showed that dopamine D1/D5 activated cAMP-PKA signal transduction pathway decreased α7nAChRs mediated whole-cell currents in the PFC (Komal et al. 2014, 2015b).

Thus, layer-specific modulation by nAChRs plays a vital role in the PFC circuitry (Poorthuis et al. 2013; Abbondanza et al. 2024).

In the basal ganglia, mainly the striatum, the source of ACh are the cholinergic interneurons (Abbondanza et al. 2024). Most of the neurons are inhibitory in nature in the striatum, where specific activation of α7nAChRs are shown to modulate dopamine neurotransmitter dynamics in this brain region (Chambon et al. 2023; Abbondanza et al. 2024). In the dorsal striatum, subcellular, synaptic, and extra-synaptic trafficking of α7 subunits containing nAChRs was shown to play a pivotal role in nicotine addiction (Pakkanen et al. 2005). In the hippocampus, α7nAChRs activation modulated the frequency of spontaneous miniature excitatory postsynaptic currents (mEPSCs) (Radcliffe and Dani 1998). A study performed by Sharma and colleagues in the hippocampus showed that presynaptically located α7nAChRs facilitated the activation of CaMKII with a concerted release of multiple vesicles on mossy fiber synapses (SharMa et al. 2008). This modulation of neuronal excitability was reported to be an action-potential independent event (SharMa et al. 2008). The complexity of the role of α7nAChRs on neural excitability are inferred mainly from pharmacologic and gene knock-out studies (Girod et al. 2000; Jones and Wonnacott 2004; Chen et al. 2006; Placzek et al. 2009; Cheng and Yakel 2015a; Eadaim et al. 2020; Assous 2021; Tsotsokou et al. 2024; Abbondanza et al. 2024). α7nAChRs activation can modify synaptic strength via both Hebbian and non-Hebbian mechanisms (Citri and Malenka 2008). It is well known that synaptic strength changes and consolidation are vital for learning and memory formation (Citri and Malenka 2008). The strengthening of synapses brought by the activation of α7 receptors at the hippocampus-medial prefrontal synapses led to the formation of associative recognition memory and induction of long-term potentiation (LTP) (Jones and Wonnacott 2004; Cheng and Yakel 2015b; Letsinger et al. 2022). On the contrary, α7nAChRs mediated enhancement of GABAergic interneurons induced long-term depression (Ji et al. 2001). α7nAChRs activation are known to enhance LTP in a PKA-dependent manner (Cheng and Yakel 2015b). An extensive regulation of glutamatergic transmission by α7nAChRs in the hippocampus is reviewed elaborately by Cheng and Yakel (Cheng and Yakel 2015b). The activation of the presynaptic located α7nAChRs in the ventral tegmental area (VTA) glutamatergic afferents is attributed to the induction of LTP (Jones and Wonnacott 2004). The expression of α7nAChRs on specific neuronal subtypes is also an important factor that determines synaptic plasticity. For example, in VTA, α7nAChRs were desensitized on dopaminergic neurons and glutamatergic afferents but activated via the application of their partial agonist, TC-7020, on the GABAergic interneurons (Maex et al. 2014). The dynamic balance brought by this receptor differs among different neural circuitry and depends on neuronal subtype and location (Yakel 2013; Verhoog et al. 2016; Letsinger et al. 2022; Sinclair and Kabbani 2023; Abbondanza et al. 2024). Thus, abundant work pinpoints the contribution of α7nAChRs in facilitating LTP and LTD (Hebbian form of synaptic plasticity) (Girod et al. 2000; Placzek et al. 2009; Yang et al. 2013; Cheng and Yakel 2015b; Verhoog et al. 2016; Assous 2021; Letsinger et al. 2022; Tsotsokou et al. 2024).

Another form of synaptic plasticity is homeostatic synaptic plasticity (HSP), which is defined as the ability of neurons to exert compensatory changes in response to altered neural activity (Stellwagen and Malenka 2006; Turrigiano 2012; Heir and Stellwagen 2020) Recently, one study showed α7nAChRs mediated homeostatic response in the Drosophila central nervous system neurons on the blockage of neural activity (Eadaim et al. 2020). α7nAChRs upregulation induced an activity-dependent homeostatic response in a Drosophila AD model (Hahm et al. 2018). This homeostatic response was also accompanied by an increased expression of the voltage-gated potassium channels (K_v_4/Shal channel) (Eadaim et al. 2020). Altogether, α7nAChRs are identified as one of the important mediators in facilitating Hebbian and non-Hebbian forms of synaptic plasticity (Chen et al. 2006; Scarr et al. 2013; Perez-Lloret and Barrantes 2016; BalLinger et al. 2016; Lewis et al. 2017; Hahm et al. 2018; Tregellas and WyLie 2019; Navarro et al. 2021; Letsinger et al. 2022; Mahmoudi et al. 2023). This unique cholinergic receptor is vital for neuronal information processing, cognition, learning, and memory function. α7nAChRs downregulation and concomitant synaptic plasticity changes get compromised in several neuropsychiatric and neurodegenerative disorders (Freedman et al. 1995; Cheng and Yakel 2015b; KoukouLi and Maskos 2015).

## Aging, Decline in α7nAChRs, and Neurodegeneration

A delicate balance between the kinases and phosphatases is vital for the cellular mechanism that regulates many ion channels and enzymes in the CNS (Khan et al. 2020). The addition or deletion of a phosphate group to its target protein reflects an exquisite mechanism for regulating protein functions by modulating either protein folding, substrate affinity, stability, or function. Protein phosphorylation is a known mechanism in regulating cell proliferation, migration, differentiation, and survival (Huganir et al. 1984, 1986). In many cases, protein phosphorylation leads to a structural change of the protein that can induce changes in interaction partners or subcellular localization (Huganir et al. 1984; Barrantes 2014, 2023). Several tyrosine kinases in the brain are known regulators of neuronal networks and synaptic transmission through the phosphorylation of nicotinic receptors (Huganir et al. 1984, 1986). 

α7nAChRs undergo phosphorylation and dephosphorylation by kinases and phosphatases, affecting their folding, assembly, trafficking, function, stability, and expression (Chrestia et al. 2021, 2023). The effect of the Src family of tyrosine kinases has been most frequently implicated in α7nAChRs function among the different kinase families that could mediate these effects on channel properties. Komal and colleagues explicitly showed that α7nAChRs undergo direct phosphorylation by Src family kinases and cAMP-dependent protein kinase (PKA). The replacement of tyrosine residue 442 (Y-442) and serine 365 (S-365), located in the M3-M4 cytoplasmic loop of the channel to alanine (Ala) abrogated the phosphorylation effect by tyrosine kinases and PKA (Komal et al. 2014, 2015a, 2022; Komal and Nashmi 2015a). The phosphorylation of Y-442 led to decreased surface expression and reduction in the single-channel conductance, while that of S-365 resulted only in decreased surface expression (Komal et al. 2014, 2015a). The effect of kinases and phosphatases on α7 receptor expression and functions are also inferred from other studies where dephosphorylation by serine–threonine phosphatases boosted α7nAChRs activity, and inhibition of these phosphatases was observed to be detrimental to receptor function (Cho et al. 2005; Charpantier et al. 2005; Chrestia et al. 2021, 2023; Jiménez-Pompa et al. 2023).

An imbalance of these enzymes impairs neuronal communication, as commonly observed in many age-related neuropsychiatric disorders, causing alterations in the neurotransmitter release, impairment in postsynaptic receptor responsiveness, and changes in the synaptic structure (Norris et al. 1998; Hsu et al. 2002; Foster 2004; Lee and Kim 2022). For example, genetic deletion of α7nAChRs led to impaired hippocampal synaptic plasticity, paralleled by increased amyloid precursor protein (APP) expression and Aβ levels (Tropea et al. 2021). In the same study, the authors argued that α7nAChRs malfunction might precede Aβ and tau pathology (Tropea et al. 2021). In a transgenic mouse model of AD activation of α7nAChRs using its specific agonist, PNU-282987 attenuated the Aβ-induced cell apoptosis, decreased the deposition of Aβ, increased the expression of synaptic-associated proteins, and maintained synaptic morphology (Wang et al. 2020b). Age-related imbalance in the regulation of synaptic function by PKA and phosphatase activities are specific to anatomic regions and neurotransmitter systems that determine synaptic efficacy (Kubanis and Zornetzer 1981; Norris et al. 1998; Hsu et al. 2002; Foster 2004). Extensive evidences reveal that a reduction in ACh synthesis, a decrease in α7nAChRs function, and depletion in cholinergic function occur in the senescent brain (Freund 1980; Gibson and Peterson 1981; Decker 1987; Tata et al. 2014; Sultzer 2018; Orlando et al. 2023). A deeper insight into the dynamics of kinases and phosphatases that cause region-specific synaptic effects, reduction in α7nAChRs number, and their synaptic availability will thus help design better therapeutics for aging and age-related neurological disorders. 

## Targeting Cholinergic Neurotransmission and α7nAChRs Activation in Brain Disorders

A breakdown in cholinergic signaling and decreased α7nAChRs function disrupts neuronal communication at the CNS synapses (Conejero-GoldBerg et al. 2008; Picciotto et al. 2012; Yang et al. 2013; Tani et al. 2015; Phenis et al. 2020; Xu et al. 2021). In several mental health disorders like SCZ, AD, PD, depression, dementia, and HD, an alteration in the cholinergic signaling is observed as one of the factors responsible for cognitive, memory, and attention impairments (OLincy et al. 2006; Smith et al. 2006; Tata et al. 2014; Perez-Lloret and Barrantes 2016; BalLinger et al. 2016; D’Souza and Waldvogel 2016; Hoskin et al. 2019; Caton et al. 2020; Iarkov et al. 2021; Terry et al. 2023). The neuroprotective benefits of α7nAChRs activation and its downstream-mediated cholinergic signaling in brain disorders are inferred mainly from *in-vivo* studies (Egea et al. 2015; Navarro et al. 2021; Xu et al. 2021; Liu et al. 2023). *In-vivo* studies performed on hemiparkinsonian and Parkinson’s rat models have shown that the activation of α7nAChRs with specific agonists like galantamine and nicotine attenuated neuroinflammation and toxin-induced loss of dopaminergic neurons (Yanagida et al. 2008; Stuckenholz et al. 2013; Quik et al. 2015; Metzner et al. 2022). The neuroprotective role of α7nAChRs is also drawn from studies performed on glial cells, where targeting these receptors alleviated nigrostriatal toxicity (Shen and Yakel 2012; Liu et al. 2015).

In AD, the hyperphosphorylation of tau and production of Aβ-amyloid plaques caused neuronal and memory loss, where targeting α7nAChRs counteracted Aβ deposition (D’Angelo et al. 2021; Hampel et al. 2021; Zhang et al. 2023). Other studies performed in cell lines and primary neuronal cultures showed that blockage of α7nAChRs via specific antagonists such as methyllycaconitine (MLA) or α-bungarotoxin (α-BTX) abrogated the neuroprotective effect against amyloid-β, glutamate, and NMDA-mediated toxicity (Kaneko et al. 1997; Dajas-Bailador et al. 2000; Takada et al. 2003; Chen et al. 2006; Yu et al. 2011; Navarro et al. 2021). Clinical studies also support the neuroprotective benefits of targeting α7nAChRs-mediated signaling in mental health disorders (Quik et al. 2015; Echeverria et al. 2016; Phenis et al. 2020; Metz and Pavlov 2021; Khattab et al. 2024; Singh et al. 2024). For example, consumption of 3-[(2,4-dimethoxy) benzylidene] anabaseine (DMXB-A), a partial agonist for α7nAChRs, improved neurocognitive defects in schizophrenic patients (OLincy and Freedman 2012).

Endogenous agonists like acetylcholine or exogenous agonists like nicotine and PNU282987 are known activators of α7nAChRs (Xu et al. 2021). α7nAChRs agonists can be divided into selective and non-selective agonists (Yang et al. 2017). Several α7 full and partial agonists like DMXBA, TC-5619, BNC375, PNU-282987, PNU120596, PHA-543613, Tropisetron, and AZD0328 have been established for reducing attention deficits and improving learning and memory (Suzuki et al. 2013; Takata et al. 2018; Wang et al. 2020b; Targowska-Duda et al. 2021; Lv et al. 2023; Yang et al. 2024; Lykhmus et al. 2024; Ongnok et al. 2024). Many compounds called positive allosteric modulators (PAMs) have been developed for α7nAChRs that do not activate α7 but enhanced ligand-induced whole-cell mediated macroscopic currents (WilLiams et al. 2011; Papke and Horenstein 2021). These compounds are divided into three subtypes and show chemical diversity, pharmacologic sensitivity, and efficacy differences (Papke and Lindstrom 2020; Papke and Horenstein 2021).

The anti-depressive role and cognitive enhancement mediated by α7nAChRs specific full agonists or partial agonists or positive allosteric modulators (PAMs) are observed in neuropsychiatric disorders like depression and SCZ (Eskildsen et al. 2014; McLean et al. 2016; Marcus et al. 2016; Neves and Grace 2018; Wang et al. 2020a; Unal et al. 2021; Targowska-Duda et al. 2021; Lv et al. 2023; Alzarea et al. 2024; Yang et al. 2024; Ongnok et al. 2024). The anti-depressive role of a common nutraceutical, Vitamin D3 (VD), in major depressive disorder (MDD), and cholinergic enhancement in HD, suggests the potential therapeutic benefits of VD supplementation in these neurological disorders (Manjari et al. 2022, 2023; Kouba et al. 2023; Skv et al. 2024). VD supplementation was shown to enhance synaptic plasticity by modulation of BDNF levels (Kouba et al. 2022, 2023). Overall, VD supplementation holds an effective therapeutic strategy for an enhancement of cholinergic signaling and α7nAChRs gene expression as demonstrated in several animal models of MDD, PD, HD, and AD (Suzuki et al. 2013; Bordia et al. 2015; Liu et al. 2015; Takata et al. 2018; Potasiewicz et al. 2020; Kouba et al. 2022, 2023; Manjari et al. 2023; Takizawa et al. 2024; Skv et al. 2024; Rabie et al. 2024; Lykhmus et al. 2024). Apart from VD, several cohort studies have emphasized the dietary intake of choline (α7nAChRs specific agonist) supplementation for cognitive benefits and sleep disorders (Yamashita et al. 2023; Huang et al. 2024, 2025). All these studies on the synthetic and natural compounds suggest that α7nAChRs may be proposed as a valuable target to treat myriad mental health conditions. However, the high desensitization kinetics, multiple non-conducting states that are rapidly interconvertible, and low probability of receptors opening in the presence of a high agonist concentration require extensive investigation into α7’s structure and function for clinical utility (Papke and Horenstein 2021).

## Vitamin D3: An AChE Inhibitor and Cognitive Enhancer in the CNS

1970s neuroscience research manifested the importance of central cholinergic mechanisms for human memory and learning (Drachman and Leavitt 1974; Petersen 1977). Cholinergic pathways undergo atrophy and cognitive decline across a range of mental illnesses like AD, PD, HD, dementia, depression, and SCZ (D’Souza and Waldvogel 2016; Hoskin et al. 2019; Tregellas and WyLie 2019; Iarkov et al. 2021; Zhao et al. 2021; D’Angelo et al. 2021; Manjari et al. 2022, 2023; Liu et al. 2023; Skv et al. 2024). The basal forebrain cholinergic neurons forms the major source for the ACh that undergoes neurodegeneration in many such neuropsychiatric disorders (Nyakas et al. 2011; SchLiebs and Arendt 2011; D’Souza and Waldvogel 2016; Björklund and Barker 2024). A recent study has shown that restoration of basal cholinergic neurons using cell-based therapy combated cognitive decline  in Parkinson's disease (Björklund and Barker 2024). Targeting drugs that can enhance cholinergic signaling appear to have a promising therapeutic role in such neuropathological conditions where they either decrease the disease progression or delay its onset (Perez-Lloret and Barrantes 2016; Sinha et al. 2020; Halder and Lal 2021; Winek et al. 2021; Papke and Horenstein 2021; Lee and Hung 2023; Manetti et al. 2023; Abdel-Magid 2024). Multiple studies have shown that intervention with cholinergic agonists targeting α7nAChRs can rescue cholinergic function and enhance memory and cognition (Nyakas et al. 2011; OLincy and Freedman 2012; Scarr et al. 2013; D’Souza and Waldvogel 2016; Hoskin et al. 2019; Iarkov et al. 2021; Lee and Hung 2023; Manetti et al. 2023; Abdel-Magid 2024; Björklund and Barker 2024).

**Fig. 3:**
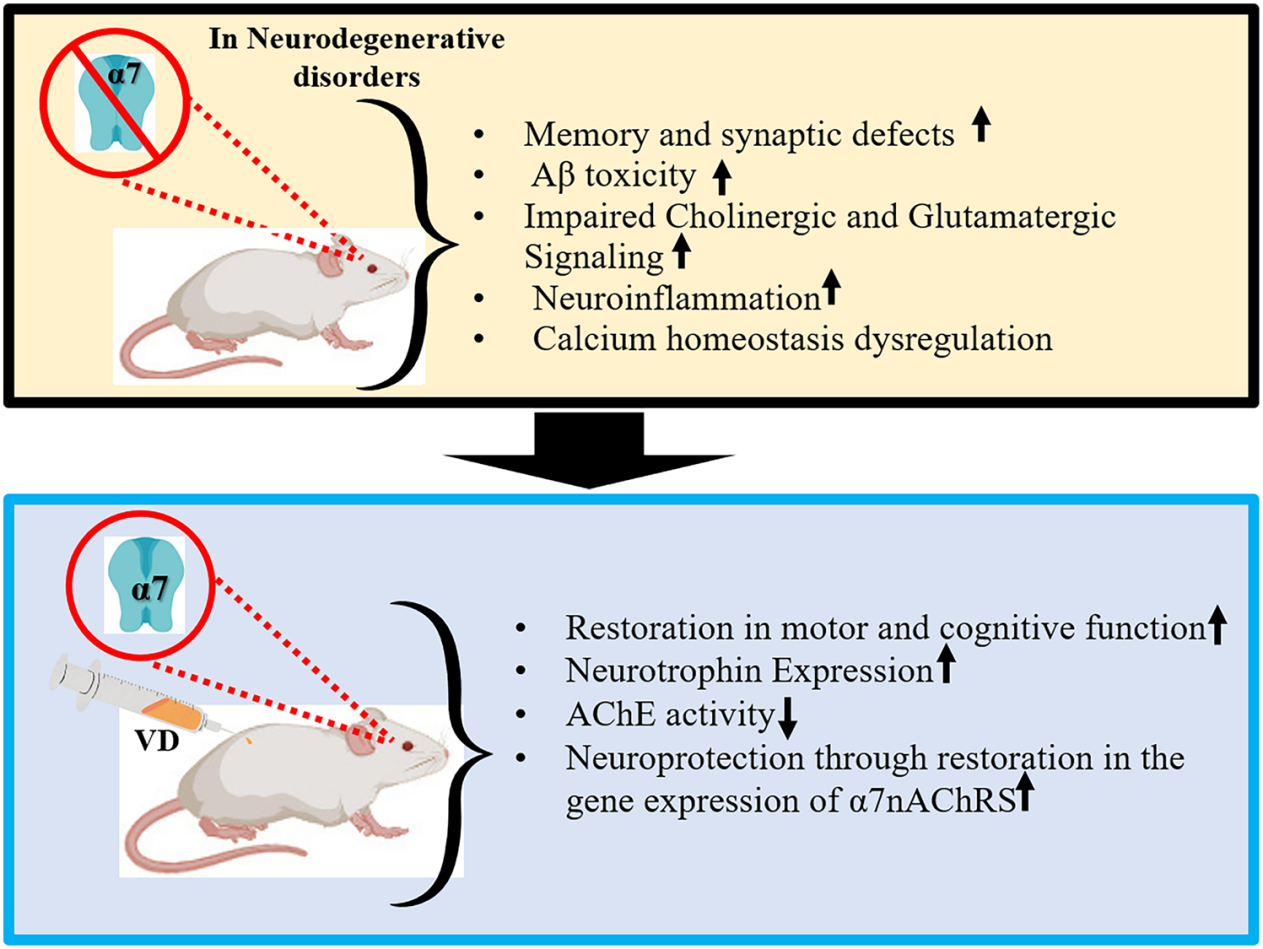
Different attributes in neurodegenerative conditions. α7nAChRs expression and function are compromised in myriad mental disorders. VD intervention enhances cholinergic activity and rescues cognition, memory, and neurotrophin expression (Conejero-GoldBerg et al. 2008; Phenis et al. 2020; Navarro et al. 2021; Xu et al. 2021; Terry et al. 2023)

In this context, the neurocognitive benefits of nutritional supplementation like VD (1alpha,25-dihydroxyVD [1α,25(OH)2D3]; cholecalciferol; VD) come from a wide range of clinical and preclinical studies (Fig. 3; Kim et al. 2006; Mohamed et al. 2015; Ishola et al. 2015; Araújo de LiMa et al. 2022; Manjari et al. 2022, 2023; Lin et al. 2022; Cui and Eyles 2022; Patel and Shah 2022; Wang et al. 2023; Sirajo et al. 2024; Skv et al. 2024). A recent finding from our lab has also demonstrated a resilience effect of VD supplementation in 3-nitropropionic acid-induced mouse model of HD (Manjari et al. 2022). A post-supplementation of 500 IU/kg of VD intervention substantially alleviated the movement abnormalities and increased α7nAChRs gene and protein expression in the striatum and the cortex. VD intervention also increased the gene expression of neurotrophins-like brain-derived neurotrophic factor (BDNF), nerve growth factor (NGF), Vitamin D receptor (VDR), and significantly subsided acetylcholine esterase activity (AChE) in HD mice (Manjari et al. 2022, 2023; Skv et al. 2024). For more details on the myriad benefits of VD in neurological disorders, please refer to our recent review by Skv et al. 2024. Evidence that parallels our work has also documented VD’s therapeutic effects in the non-neuronal cells of the central and peripheral nervous system, where VD’s intervention increased VDR, NGF, and Neurotrophin-3 (NT-3) gene expression (Adams and Gacad 1985; Neveu et al. 1994b, a; Cornet et al. 1998).

A neuro-regenerative and neuroprotective role of VD comes from various studies where VD treatment improved myelination in the injured neurons of the peripheral nervous system (Chabas et al. 2013; Montava et al. 2015). Chabas and group showed that VD treatment to Schwann cell or DRG cultures enhanced the gene expression of key genes like Igf1, Metrn, Limk1, Ulk2, Prx, Tspan-2, Spp1 that are known to play essential roles in axogenesis and myelination (Chabas et al. 2013). In an experimental rat model of peripheral neuropathy, VD administration promoted structural and functional recovery of injured peripheral neurons (Erdem et al. 2023). In the non-neuronal cells of the CNS, VD-mediated signaling was demonstrated to enhance an anti-inflammatory environment and increased the production of anti-inflammatory cytokines, with a shift in the phenotype of activated microglia from M1 to M2(CaLvello et al. 2017; Cui et al. 2019; Lee et al. 2020). In primary mouse astrocyte cultures, Isabelle and colleagues showed that VD induction increased mRNA expression of NGF and neurotrophin-3 (NT-3, Neveu et al. 1994b). Another study performed again in primary mouse astrocyte cultures showed the anti-aging potential of a combination of lipoic acid and VD (MoLinari et al. 2019). The combination therapy alleviated oxidative stress, ROS levels, cytochrome C expression, prevented intracellular iron accumulation, and decreased p53 activity in astrocytes that boosted mitochondrial health (MoLinari et al. 2019).

Recently, Duygu Gezen-Ak and colleagues reflected the importance of VD-VDR on mitochondrial health and biogenesis, where the authors argued that disruption of VD-VDR homeostasis can lead to mitochondrial dysfunction and neurodegeneration (Gezen-Ak et al. 2023). The correlation between the increased risk of development and adult VD deficiency and neurodevelopmental disorders has been provided extensively by the Eyles group for the last few decades (Eyles et al. 2007, 2013, 2024; Eyles 2020; Cui and Eyles 2022). It is now known that VD shows its biological effects mainly by binding to Vitamin D receptor (VDR, Eyles et al. 2024; Skv et al. 2024). VD modulates the brain’s calcium (Ca2+) homeostasis and ameliorates excessive excitotoxicity damage to neurons by downregulating the L-type voltage-gated ion channel (L-VGCC) expression. There are also compelling evidences that reflects a strong association between L-VGCC gene expression and Vitamin D signaling (Brewer et al. 2001, 2006). For example, in primary hippocampal neurons, it was shown that VD induction decreased the expression of the L-VGCC pore-forming domain and conferred neuroprotection (Brewer et al. 2001, 2006). In these studies, VD was also observed to increase the extracellularly derived uptake of Ca2+ levels in a concentration-dependent manner. This stimulatory effect depended on L-VGCC activity, intracellular calcium release, K+ and Cl- ion channels, and the modulation of Na+/K+-ATPase activity. It is interesting to note that the changes in L-VGCCs represent the most replicated genetic abnormality in psychiatric conditions such as SCZ and autism (Heyes et al. 2015; Liao and Li 2020; Wang et al. 2022). VD-VDR signal involves several different protein kinases, including PKA, Ca2+ /calmodulin-dependent protein kinase (CaMKII), mitogen-activated kinase (MAPK), and phosphatidylinositol 3-kinase (P13K) (Haussler et al. 1998; Gezen-Ak et al. 2011; Dursun and Gezen-Ak 2017; Bao et al. 2020; da SiLva Teixeira et al. 2020; Wang et al. 2023; Lasoń et al. 2023; Skv et al. 2024). Exploring all these myriad signal mechanisms and dynamic changes in kinases and phosphatases brought by VD-VDR interaction and its effect on α7nAChRs signaling (Fig. 2) may illuminate potential therapeutic targets for neurological disorders and their treatment.

## Challenges and Limitations

α7nAChRs are recognized as one important ligand-gated ion channel (LGIC) that forms a vital component in the brain's cholinergic system and modulates neurotransmission (Broide and LesLie 1999; Berg and Conroy 2002). The extraordinary calcium permeability and fast excitatory synaptic neurotransmission make α7nAChRs distinct from other nAChRs. Dysregulation of these receptors can impair the electrochemical signal transduction system in the nervous system, as observed in several neuropsychiatric and neurodegenerative disorders, highlighting the need for a comprehensive understanding of their regulation and function (Quik et al. 2015; Ma and Qian 2019; Tregellas and WyLie 2019; Lee and Hung 2023; Manjari et al. 2023).

Clinical trials with α7nAChR agonists have shown promising results in improving cognitive deficits in SCZ (Yang et al. 2017). However, none of the drugs targeting α7 have passed clinical trials, mainly due to adverse side effects and high receptor desensitization kinetics (Tregellas and WyLie 2019; Lee and Hung 2023). This property of the receptor poses a challenge to the development of therapeutic compounds for mental health disorders. Several PAM identifications, however, have opened a new era of computer-aided structural investigation for α7nAChRs that may hold exceptional therapeutic potential for targeting this receptor in neuropsychiatric disorders (Burke et al. 2024).

Several cholinesterase-inhibiting drugs like donepezil, galantamine, or rivastigmine are currently prescribed for AD, but each has its own demerits and limitations (Rogers and Friedhoff 1996; Sharma 2019). There is a dire need to explore in detail the underlying mechanism by which natural nutraceutical like Vitamin D3 (VD) decreases acetylcholine esterase (AChE) activity (Fig. 2), along with its anti-inflammatory, antioxidant, and neuroprotective benefits in neurological disorders (Manjari et al. 2022; Skv et al. 2024). Presently, the efficacy of this nutraceutical in the field of neurodegenerative disorders has been controversial. Still, given the ionotropic and non-ionotropic function of α7nAChRs, VD-mediated signaling and its interaction with α7nAChR regulation represent a promising area for future investigations and therapeutic interventions. Since α7nAChRs undergo significant downregulation in various mental disorders, causing cognitive deficits and other symptoms, a multidrug-conjugated approach utilizing VD and deeper insights into α7 structure–function analysis may lead to better remedies in the field of neurodegenerative and neuropsychiatric disorders (Tregellas and WyLie 2019; Potasiewicz et al. 2020; Phenis et al. 2020; Burke et al. 2024; Gajendra et al. 2024; ZHuang et al. 2024).

## Data Availability

No datasets were generated or analysed during the current study.

## Abbreviations

VD3: Vitamin D3 or VD
ACh: Acetylcholine
PAM: Positive allosteric modulator
CNS: Central nervous system
HD: Huntington’s disease
PD: Parkinson’s disease
AD: Alzheimer’s disease
SCZ: Schizophrenia
nVDR: Nuclear Vitamin D Receptor
VDRE: Vitamin D response element
1-alpha-25-dihydroxyvitamin D3: 1-alpha-25-dihydroxyvitamin D3 (1ɑ,25-(OH)2D3) or calcitriol
25-hydroxyvitamin D3: 25(OH)D3 or calcidiol
GPCR: G-protein-coupled receptors
MAPK: Mitogen-activated protein kinases
ERK: Extracellular signal-regulated kinase
AKT: Protein kinase B
cAMP: Cyclic adenosine monophosphate
PKA: Protein kinase A
IP3: 1,4,5-Trisphosphate
Ca2+: Calcium
RXR: Retinoid X receptor
CREB: cAMP response element-binding protein
IL-10: Interleukin 10
IL-4: Interleukin 4
BDNF: Brain-derived neurotrophic factor
NGF: Nerve growth factor
GDNF: Glial cell line-derived neurotrophic factor
NT-3: Neurotrophin-3
NT-4: Neurotrophin-4
